# Supplementary material for: Experimental investigation of bio-improved laterite for use as a subgrade material in road construction
Source: PLoS One. 2025 Oct 29;20(10):e0335062. doi: 10.1371/journal.pone.0335062 (PMC12571301; doi:10.1371/journal.pone.0335062)
Supplement: Attachment — Submitted filename: Response to Reviewers (2).docx [file pone.0335062.s002.docx]

Dear Editor,

Below are responses to each reviewer's comments. I would also like to make changes to my financial disclosure.

Kind regards

Sani Reuben O ***PhD***

*Department of Civil Engineering*

*College of Engineering*

*Covenant University, Ota,*

*Nigeria.*

| S/N | Comment | Response |
| --- | --- | --- |
| 1 | Please ensure that your manuscript meets PLOS ONE's style requirements, including those for file naming. The PLOS ONE style templates can be found at [https://journals.plos.org/plosone/s/file?id=wjVg/PLOSOne_formatting_sample_main_body.pdf](https://track.editorialmanager.com/CL0/https:%2F%2Fjournals.plos.org%2Fplosone%2Fs%2Ffile%3Fid=wjVg%2FPLOSOne_formatting_sample_main_body.pdf/1/010f0193dd8a76aa-7858df69-0ade-40bb-bcc7-e43b03803da8-000000/8lVwnzxViDTfFs364U0Ao9EiqFYhxLdf9ACSnWgur6k=190) and [https://journals.plos.org/plosone/s/file?id=ba62/PLOSOne_formatting_sample_title_authors_affiliations.pdf](https://track.editorialmanager.com/CL0/https:%2F%2Fjournals.plos.org%2Fplosone%2Fs%2Ffile%3Fid=ba62%2FPLOSOne_formatting_sample_title_authors_affiliations.pdf/1/010f0193dd8a76aa-7858df69-0ade-40bb-bcc7-e43b03803da8-000000/AMINUAl9RZsEl3ypWrFPVC9F5Wm9glFL_BjAuybW1yc=190) | Manuscript formatted to meet PLOS ONE style templates. |
| 2 | In your Methods section, please provide additional information regarding the permits you obtained for the work. Please ensure you have included the full name of the authority that approved the field site access and, if no permits were required, a brief statement explaining why. | Additional information provided in the Methods section |
| 3 | We note that your Data Availability Statement is currently as follows: [All relevant data are within the manuscript and its Supporting Information files] | The submission contains all raw data required to replicate the study results. |
| 4 | We note that Figures S2 and S3 includes an image of a participant in the study.As per the PLOS ONE policy ([http://journals.plos.org/plosone/s/submission-guidelines#loc-human-subjects-research](http://track.editorialmanager.com/CL0/http:%2F%2Fjournals.plos.org%2Fplosone%2Fs%2Fsubmission-guidelines%23loc-human-subjects-research/1/010f0193dd8a76aa-7858df69-0ade-40bb-bcc7-e43b03803da8-000000/0KUH2hrEhg3zP3nw78fWFOIYm_0NIH78x7ZODSh6bhg=190)) on papers that include identifying, or potentially identifying, information, the individual(s) or parent(s)/guardian(s) must be informed of the terms of the PLOS open-access (CC-BY) license and provide specific permission for publication of these details under the terms of this license. | Participants were removed from Figure S2. The consent form filled have been filled by participants is in Figure S3. |
| 5 | Please upload a copy of Figures 1 to 15, to which you refer in your text on pages 9,10,11,12,13,14,15,16,18,19 and 20. If the figure is no longer to be included as part of the submission please remove all reference to it within the text. | Figures were uploaded as supporting documents according to PLOS ONE requirements. |
| 6 | The rationale for selecting Bacillus Anthracis over other urease-positive bacteria is unclear. Include a justification, addressing potential environmental or safety implications of using this bacterium. | The Taguchi analysis provides reasons for selecting each parameter data point (bacteria type: Bacillus Anthracis inclusive).  Though the use of MICP for soil improvement has been researched extensively to be environmentally friendly, and justification statements (lines 58 – 63) already in introduction section https://doi.org/10.1016/j.sandf.2020.07.003  https://doi.org/10.1061/(asce)mt.1943-5533.0003036.  The byproducts of the MICP reaction have been discovered to produce ammonia, which is harmful at concentrated doses. Also, most organisms used with the bacteria “Bacillus Anthracis” used for these research work can be harmful to humans. https://en.wikipedia.org/wiki/Bacillus_anthracis. |
| 7 | Section 2.3 shows a flowchart of the method of the work. I prefer to move this section to be the beginning of section 2 so the reader can see what steps the authors have taken and then explain each step in the following subsections. | Sectio moved to the beginning of section 2 |
| 8 | Figure 7 requires more explanation. What do the numbers on the horizontal and vertical axes refer to? | More explanations have been provided for Figure 7. Explanations are also provided for numbers on vertical axes, and the section on the parameter selection process contains explanations of numbers on horizontal axes. |
| 9 | I could not find table 4 | Table 4 included |
| 10 | The introduction discusses the environmental benefits of MICP but lacks a comparative analysis with traditional soil stabilization methods like lime or cement. Adding this would strengthen the case for MICP. | This research focuses on finding solutions to the global push for net-zero carbon dioxide emissions by finding replacements for cement, lime, and other common soil improvement materials, i.e., environmental benefits/impacts in relation to road construction.  A detailed comparative analysis would not only entail environmental benefits/impacts but durability, implementation complexity, soil type suitability, strength improvement, cost comparison, permeability reduction, reaction/setting time, and implementation complexity. |
| 11 | The manuscript reports significant improvements in CBR and UCS values but does not clearly explain why the improvement plateaus at higher bacterial concentrations. This phenomenon should be discussed in detail. | Discussions have been added to the manuscript: "Although higher bacterial concentrations have been reported to enhance higher calcite precipitation, leading to better soil improvements, contrary results, like those obtained in this manuscript, have shown that the highest percentage of calcite is being obtained at a low concentration”. Citations are also provided. |
| 12 | While regression analysis results are included, confidence intervals and statistical significance for model coefficients are missing. Incorporating these would improve the credibility of the statistical approach. | Confidence interval of 95 % and statistical significance of 0.05 presented and explained in statistical section. |
| 13 | The field testing section briefly mentions compaction methods but does not provide sufficient details on replication or error margins, which are necessary for validating field-to-lab result comparisons. | The focus was more on very relevant soil properties in relation to road construction, such as strength improvements to carry traffic load, permeability, shrink and swell tendencies, etc, but the core cutter test to address this point was performed and results were presented in Table 9 |
| 14 | Key citations are missing in the manuscript. Please include references to support the claims and findings. Consider adding the following citations at suitable sections: a. <https://doi.org/10.1007/978-981-15-9554-7_10> b. <https://doi.org/10.1007/978-981-16-6557-8_75> c. <https://doi.org/10.1007/978-981-16-6557-8_78> d. <https://doi.org/10.22044/jme.2023.13018.2361> e. <https://doi.org/10.1080/15440478.2020.1848699> | Citations added |
| 15 | Figures presenting test results (e.g., load-penetration curves) lack consistent scales and annotations, making it difficult to interpret trends. Ensure uniform formatting and include detailed captions. | Uniform formatting carried out |
| 16 | The study demonstrates an 80% improvement in CBR, but the final values remain below the threshold typically required for road subgrade materials. The conclusion should reflect this limitation more explicitly. | Limitation presented in conclusion sections |
| 17 | The methodology section does not explain why certain parameters (e.g., 3.0 × 10^8 cells/ml concentration) were chosen over others. A brief explanation or citation to prior studies would address this gap. | The reason for using the Taguchi design of experiment and analysis in this study was to address this question. It helped to reduce the vast number of experiments that would have been carried out if a full factorial experimental design had been considered. |
| 18 | The study’s practical implications are discussed but lack a cost-benefit analysis comparing MICP treatment with traditional methods. Adding this analysis would provide a more comprehensive perspective. | The authors agree with this point but are of the opinion that including a detailed and acceptable cost-benefit analysis in relation to road construction involves a lot of data and results entailing a complete manuscript, hence the focus and scope of the manuscript on the experimental investigation |
| 19 | The use of Bacillus Anthracis in MICP for road construction is novel, but the manuscript does not address potential scalability or challenges in deploying this approach at larger scales, which should be included in the discussion. | Scalability and challenges in deploying this approach at larger scales would be explained better if the manuscript addressed comment 18. However, a common challenge from various research works that have tried to upscale MICP to the field has been the very high cost of chemical materials. Most research works have found that MICP is quite costly to implement in the field  <https://doi.org/10.1016/j.cscm.2023.e02086>  <https://doi.org/10.1017/cbo9780511802409.013>  <https://doi.org/10.1080/01490451.2016.1225866>  <https://doi.org/10.1016/j.micres.2016.03.010> |
