# Supplementary material for: Experimental investigation of bio-improved laterite for use as a subgrade material in road construction
Source: PLoS One. 2025 Oct 29;20(10):e0335062. doi: 10.1371/journal.pone.0335062 (PMC12571301; doi:10.1371/journal.pone.0335062)
Supplement: Attachment — Submitted filename: Response to Reviewers with cover letter.docx [file pone.0335062.s003.docx]

Dear Editor,

Below are responses to each reviewer's comments.

I would also like to make changes to my financial disclosure.

Kind regards

Sani Reuben O ***PhD***

*Department of Civil Engineering*

*College of Engineering*

*Covenant University, Ota,*

*Nigeria.*

| S/N | Comment | Response |
| --- | --- | --- |
| 1 | Suppose the authors have adequately addressed your comments raised in a previous round of review and you feel that this manuscript is now acceptable for publication. In that case, you may indicate that here to bypass the "Comments to the Author" section, enter your conflict of interest statement in the "Confidential to Editor" section, and submit your "Accept" recommendation.  Reviewer #1: (No Response)  Reviewer #2: All comments have been addressed | *Acknowledged with thanks* |
| 2 | Is the manuscript technically sound, and do the data support the conclusions? The manuscript must describe a technically sound piece of scientific research with data that supports the conclusions. Experiments must have been conducted rigorously, with appropriate controls, replication, and sample sizes. The conclusions must be drawn appropriately based on the data presented.  Reviewer #1: Partly  Reviewer #2: Partly | Partial acceptance of the manuscript's technical aspects may be due to its strong geotechnical engineering context and its target audience, who are practitioners in the geotechnical engineering field.  The response below should be helpful towards a complete acceptance.  Not neglecting the global standards of writing quality journal papers, the manuscript addressed key technical concerns that would arise when geotechnical practitioners read the manuscript.   1. *What's the novelty of the research?* *Ans. Limited field and laboratory investigations have investigated the applicability of MICP in road construction due to the general notion that MICP is only applicable to soils with optimum grain size between 50 and 400 µm, and the common MICP treatment methods to achieve homogeneity of the improved soil do not apply to road construction. Laterite soil, the material used for this research, does not fully fall within this optimum.* 2. *Why is the research necessary? The global drive to reduce greenhouse gas (GHG) emissions and air pollution associated with the production of cement and other cement-related materials used during construction.* 3. *How was the identified problem solved so that it can be replicated by any geotechnical engineer, not just in the laboratory, but in the field?*  - *The first thought, to any geotechnical engineering practitioner when addressing this question, would be, what parameters would influence this MICP process in relation to road construction? The manuscript identified six vital parameters.* - *Second thought, at what range would these parameters be applied? The authors identified the need to adopt an optimisation process to limit the number of experiments while producing optimal results, and also, to answer common review questions on "what informs the adoption of parameter ranges used for research works?" Hence, the adoption of the Taguchi design of experiments. (The authors acknowledge the vast optimisation techniques available for design of experiments and optimisation, but the scope was limited to the Taguchi method, thereby raising an area for further research works using other methods.) Detailed procedures on this were provided in the manuscript at the methods section, and where not provided, references were given to ease replicability. Detailed and complete results applicable to this research were provided and discussed; Table 3 and Figure 7 (S7. Fig). See supporting information. And conclusions drawn from these results* - *Third thought: Does the soil actually need improvement? i.e., further justifying the work. Following the detailed procedure provided in the manuscript at the methods section, and where not provided, laboratory codes are cited for replicability. Standard required geotechnical tests were carried out, and results were presented in Tables 2 and 3, Figures 5 and 6, and discussions were provided where the AASTHO and USCS classifications conclusively allay this thought.* - *Fourth thought: What important geotechnical properties are investigated in relation to road construction? (Strength? Permeability? and susceptibility to cracks; weight and volume changes?). Were findings discussed and compared with other researchers' results? Are there available standard values and requirements that must be met for an improved soil to be used for road construction? Ans. Detailed and complete results, discussions, and conclusions regarding these key geotechnical aspects were provided in the manuscript.* - *Other thoughts that were addressed, that require detailed and complete results, discussions relating research findings to those from other research works, and conclusions based on research findings in relation to these thoughts include:* - *Since the work involves a reaction, was the morphology investigated? Ans. the Authors presented detailed results, discussions and conclusions on this using the SEM and FTIR analysis.* - *Was there any form of modelling to enhance the quality of the research by providing a framework to organize information, identify key relationships between variables, predict future outcomes, test hypotheses under controlled conditions, and visualize complex systems that are difficult to observe directly? Ans. Using the Minitab software to run the Taguchi optimization process and a regression analysis, detailed and complete results, which included models, were presented, discussed, and supported by statistical tables, and conclusions were drawn based on findings.* |
| 3 | Has the statistical analysis been performed appropriately and rigorously?  Reviewer #1: N/A  Reviewer #2: Yes | *Acknowledged with thanks* |
| 4 | 4. Have the authors made all data underlying the findings in their manuscript fully available?  The [PLOS Data policy](http://track.editorialmanager.com/CL0/http:%2F%2Fwww.plosone.org%2Fstatic%2Fpolicies.action%23sharing/1/010f019786df13fd-0d430724-0264-4d24-94c5-df56f1fd7cda-000000/XGGo3DhbirbzjAVI43mWYO_mWUbXH5Q0FVhtVLnhX20=216) requires authors to make all data underlying the findings described in their manuscript fully available without restriction, with rare exception (please refer to the Data Availability Statement in the manuscript PDF file). The data should be provided as part of the manuscript or its supporting information, or deposited to a public repository. For example, in addition to summary statistics, the data points behind means, medians and variance measures should be available. If there are restrictions on publicly sharing data—e.g. participant privacy or use of data from a third party—those must be specified.  Reviewer #1: No  Reviewer #2: Yes | *All essential and detailed relevant data underlying findings in the manuscript were made available and presented in the form of tables and charts, from where discussions were made and conclusions drawn.*  *A possible reason for a “No” from Reviewer #1 might be that figures were uploaded as supporting documents according to PLOS ONE requirements, hence cannot be seen on the main manuscript.* |
| 5 | Is the manuscript presented in an intelligible fashion and written in standard English?  PLOS ONE does not copyedit accepted manuscripts, so the language in submitted articles must be clear, correct, and unambiguous. Any typographical or grammatical errors should be corrected at revision, so please note any specific errors here.  Reviewer #1: Yes  Reviewer #2: Yes | *Acknowledged with thanks* |
| 6 | Review Comments to the Author  Please use the space provided to explain your answers to the questions above. You may also include additional comments for the author, including concerns about dual publication, research ethics, or publication ethics. (Please upload your review as an attachment if it exceeds 20,000 characters)  Reviewer #1: Dear Author,  I could not find the figures in the manuscript or in a separated file Could you please attach them  Reviewer #2: The manuscript presents valuable insights; however, it would benefit significantly from a thorough English language revision to enhance clarity, coherence, and overall readability. Several sentences are grammatically incorrect or awkwardly phrased, which may hinder the reader's understanding of the content. The use of technical terms is sometimes inconsistent, and transitions between sections can be improved to ensure a smoother flow of ideas. Additionally, the manuscript contains typographical errors and improper punctuation in multiple places. It is recommended that the authors seek assistance from a professional English editing service or a native English-speaking colleague with academic writing experience. Improving the manuscript's language quality will not only make the arguments more persuasive but also increase its impact and accessibility to a broader academic audience. Once the language issues are addressed, the manuscript's scientific merit and contributions will be more effectively communicated | Reviewer 1,  *Pace corrected Figures 1 - 15 where attached as a separate Zip file titled "Supporting Information - Compressed/ZIP File Archive" in accordance to PLOS ONE requirements, as supporting information in a Zip file which was might have not been forwarded along to you for review.*    Reviewer 2:  *A fully subscribed Grammarly package has been used to proofread the entire manuscript, addressing raised concerns* |
